# Growth Abnormalities as a Risk Factor of Adverse Neonatal Outcome in Hypertensive Pregnancies—A Single-Center Retrospective Cohort Study

**DOI:** 10.3390/children8060522

**Published:** 2021-06-19

**Authors:** Anna Kajdy, Stepan Feduniw, Jan Modzelewski, Dorota Sys, Dagmara Filipecka-Tyczka, Katarzyna Muzyka-Placzyńska, Paweł Kiczmer, Bartłomiej Grabowski, Michał Rabijewski

**Affiliations:** 1 Department of Reproductive Health, Centre of Postgraduate Medical Education, 01-004 Warsaw, Poland; sfeduniw@cmkp.edu.pl (S.F.); jmodzelewski@cmkp.edu.pl (J.M.); dsys@cmkp.edu.pl (D.S.); dagamra.filipecka-tyczka@cmkp.edu.pl (D.F.-T.); katarzyna.muzyka-placzynska@cmkp.edu.pl (K.M.-P.); mirab@cmkp.edu.pl (M.R.); 2 Department and Chair of Pathomorphology, Faculty of Medical Sciences in Zabrze, Medical University of Silesia, 40-055 Katowice, Poland; pawel.kiczmer@protonmail.com; 3 Faculty of Medicine, Lazarski University, 02-662 Warsaw, Poland; bartlomiej.grabowski@lazarski.pl

**Keywords:** pregnancy hypertension, prepregnancy hypertension, preeclampsia, growth chart, small for gestational age, large for gestational age, neonatal growth restriction, composite neonatal outcome, intrauterine death, neonatal death

## Abstract

(1) Background: Hypertensive disorders of pregnancy (HDP) include gestational hypertension (GH), chronic hypertension (CH), preeclampsia (PE), and preeclampsia superimposed on chronic hypertension (CH with PE). HDP is associated with several short and long-term perinatal and neonatal complications, such as newborn growth restriction and death. This study aimed to establish the association between HDP, newborn growth abnormalities, and neonatal outcome. (2) Methods: This is a single-center retrospective cohort study of 63651 singleton deliveries. (3) Results: Univariate analysis showed a significantly increased risk of intrauterine and neonatal death associated with maternal hypertension and growth disorders. There were differences between growth charts used, with the highest risk of stillbirth for SGA defined by the Intergrowth chart (OR 17.2) and neonatal death for newborn growth restriction (NGR) based on Intergrowth (OR 19.1). Multivariate analysis showed that NGR is a stronger risk factor of neonatal death than SGA only. (4) Conclusions: HDP is significantly associated with growth abnormalities and is an independent risk factor of adverse outcomes. The presence of newborn growth restriction is strongly associated with the risk of neonatal death. The choice of growth chart has a substantial effect on the percentage of diagnosis of SGA and NGR.

## 1. Introduction

Hypertensive disorders of pregnancy (HDP) include gestational hypertension (GH), chronic hypertension (CH), preeclampsia (PE), and preeclampsia superimposed on chronic hypertension (CH with PE). It complicates 4–8% of all pregnancies [1,2]. HDP is associated with several short and long-term perinatal and neonatal complications. The main being increased risk of intrauterine and neonatal death. The primary risk factors of mortality are birth weight and gestational age at delivery and are mainly attributed to early onset hypertensive disorders [3].

In 2016 an international consensus on the definition of fetal growth restriction was published, followed by an international consensus on neonatal growth restriction [4]. Neonatal growth restriction has been defined as birth weight less than the third centile on population-based or customized growth charts or a combination of at least 3 out of 5 of the following: birth weight < 10th centile on population-based or customized growth charts, head circumference < 10th centile, length < 10th centile, prenatal diagnosis of fetal growth restriction and presence of HDP in the mother [5].

The choice of growth chart has a significant impact on the diagnosis of growth abnormalities. Reference birthweight population charts are a source of bias, especially for preterm deliveries. On the other hand, international standard growth charts identify a smaller number of neonates below the 10th centile for a given population [6]. Customized charts consider maternal height, weight, and parity but have not been found to identify at-risk pregnancies better than population charts [7,8,9]. The best charts are charts constructed prospectively on low-risk populations after excluding risk factors of abnormal growth. The construction of these charts is time- and money-consuming. Many studies have been conducted to compare the efficacy of growth charts in predicting abnormal outcomes, but not specifically in a large population of pregnancies complicated by hypertensive disorders [8,9,10,11].

Thus far, only one study has attempted to validate the consensus definition of fetal growth restriction, comparing it to FGR defined as an estimated fetal weight below 10th centile by Hadlock. The consensus definition better identifies at-risk neonates than estimated fetal weight below 10th centile by Hadlock [10]. The international consensus definition of NGR has not yet been validated. Few studies have looked at the relationship between the different spectrums of HDP, definitions of growth abnormalities (SGA, LGA, NGR), and neonatal outcome [12,13,14]. 

The primary aim of this study was to establish an association between different hypertensive disorders of pregnancy, growth abnormalities, and neonatal outcomes. The secondary aim was to confirm previously established risk factors and identify new risk factors of intrauterine fetal death, neonatal death, and composite neonatal outcome. 

## 2. Materials and Methods

### 2.1. Study Design

This is a single-center retrospective cohort study. Strobe guideline for cohort studies was used to ensure proper reporting of data and outcomes [15]. The study has received approval from the Bioethics Committee of the Centre of Postgraduate Medical Education (No. 101/PB/2019). It was a retrospective analysis of anonymized data. Therefore, individual patient consent was not needed. 

### 2.2. Setting, Participants, and the Study Size

An anonymous retrospective database of all deliveries between 2010 and November 2020 was created using electronic patient records of Saint Sophia’s Hospital in Warsaw, Poland, a tertiary hospital, with the largest number of deliveries per year. All singleton deliveries were eligible except those with major congenital anomalies (as described in EUROCAT Guide 1.4) or abnormal karyotype neonates [16,17]. Modest exclusion criteria were used. Therefore, the risk of selection bias in this study is low. The dataset was created using electronic medical records collected by professional medical personnel. Thus, there is no recall bias. Additionally, the dataset was rigorously cross-checked for inconsistencies, such as differences in the medical diagnosis, the treatment used, and the ICD codes used, and any detected were verified. 

The study group was subdivided into four subgroups: Group 1: gestational hypertension (GH), Group 2: chronic hypertension (CH), Group 3: preeclampsia (PE), and Group 4: chronic hypertension with superimposed preeclampsia (CH with PE). Definition of GH, CH, and CH with PE was made according to the International Society’s for the Study of Hypertension in Pregnancy guidelines [2]. Preeclampsia was defined as systolic blood pressure > 140 mm Hg and/or the diastolic blood pressure > 90 mmHg on at least two occasions four hours apart developing after 20 weeks gestation with significant proteinuria (>300 mg in 24 h, or two readings of at least ++ on dipstick analysis of midstream or catheter urine specimen, if no 24 h collection is available) or the presence of fetal growth restriction. Diagnosis of superimposed PE on chronic hypertension requires the development of significant proteinuria after 20 weeks gestation in a previously non-proteinuric woman [2].

Birth weight was expressed in centiles for gestational age at delivery. Three birthweight charts were compared: Intergrowth, Fenton, and the Polish Reference chart. [6,18,19] The characteristics of the birth-weight charts is shown in Table 1. SGA was defined as birth weight below the 10th centile, AGA as birthweight between the 10 and 90th centile, and LGA as birthweight above the 90th centile [20,21].

Fetal growth restriction diagnosis was based on medical records of prenatal diagnosis based on ultrasound assessment defined as an estimated fetal weight below 3rd centile or estimated fetal weight between 3 and 10th centile by Hadlock and abnormal Doppler indices (UtA, UA, MCA, CPR). This information was needed for defining neonatal growth restriction (NGR). NGR was based on the international consensus definition: birth weight less than the third percentile on population-based or at least 3 out of 5 of the following: birth weight < 10th percentile on population-based, head circumference < 10th percentile, length < 10th percentile, prenatal diagnosis of fetal growth restriction, maternal pregnancy information regarding hypertension and/or preeclampsia [5]. The Fenton and Intergrowth charts were used to define neonatal growth restriction. 

### 2.3. Variables and Measurement

Primary outcomes were defined using the CROWN initiative, with additional outcomes defined for this study [22]. The primary results were stillbirth (SB), neonatal death (ND), and composite neonatal outcome (CNO), a combination of all secondary outcomes. The secondary outcomes were cesarean section for fetal distress, instrumental vaginal delivery for fetal distress, respiratory distress syndrome, intraventricular hemorrhage grade III or IV, neonatal sepsis, periventricular leukomalacia, confirmed seizures, and Apgar Score < 8 at 5 min of observation.

### 2.4. Risk of Bias

This was a retrospective study in which there is an a priori higher risk of bias. The dataset regarding diagnosis and outcomes was built using electronic patient records. Records were cross-checked for consistency of ICD-10 codes and descriptive diagnosis to reduce bias. Prenatal diagnosis of fetal growth restriction was also based on electronic patient records and not an ultrasound database. Neonatal growth restriction (NGR) could not be defined according to Polish reference charts due to a lack of charts regarding length and head circumference. All these biases and limitations will be discussed in the discussion section of the article.

### 2.5. Statistical Analysis

All statistical analyses were performed using STATISTICA 13.3 version (StatSoft, Inc.). Data were demonstrated as average ± standard deviation (SD). The relationship of quantitative variables across the groups was calculated using the t-student test and simple ANOVA, and consequent Scheffe posthoc analysis. The significance of qualitative variables was calculated using the chi-square test. A logistic model was performed using a backward stepwise validation method to determine predictors of factors influencing the risk of neonatal death, stillbirth, and CNO. Both uni- and multivariate model results were presented as odds ratios with a 95% confidence interval. In all calculations, significant values were assessed for *p*-value ≤ 0.05.

## 3. Results

A total of 63,651 singleton deliveries were included. They were divided into four study groups according to the type of hypertensive disorder: Group 1: GH (*n* = 2061), group 2: CH (*n* = 542), group 3: PE (*n* = 435), and Group 4: CH with PE (*n* = 720). The Control group consisted of 60,540 women without any HDP. The baseline characteristics of the studied population is shown in Table 2.

Growth restriction in the newborn was based on a consensus definition [5]. There were differences in the percentage of neonates defined as growth-restricted depending on the growth chart used. Fenton yielded 29% of SGA neonates to be growth restricted to 40% of those defined by the Intergrowth chart. Besides birth weight below the third centile, the additional criteria increased the number of defined neonates by 47.6% for Intergrowth and 23.4% for Fenton. (Table 3).

Logistic regression models were performed to evaluate maternal hypertension’s influence on the risk of stillbirth and neonatal death. Univariate analysis showed a significantly increased risk of intrauterine and neonatal death associated with maternal hypertension and growth abnormalities. There were differences between growth charts used, with the highest risk of stillbirth for SGA defined by the Intergrowth chart (OR 17.2) and ND for NGR based on Intergrowth (OR 19.1). All logistic regression data are presented in Table 4. The statistically significant results are bolded. In the next step, a multivariate model was performed using the stepwise backward validation method. 

The multivariate model showed that the strongest risk factor for ND was gestational age < 34 (OR 166) and being SGA on the Polish reference growth chart (OR 6.78). To exclude the strong effect of prematurity, an additional model was performed, excluding gestational week. That model showed that the newborn’s maternal hypertension and growth restriction are independent risk factors increasing neonatal death (Table 5). Multivariate analysis showed that NGR is a stronger risk factor of neonatal death than SGA only. In the multivariate model excluding gestational age, the Polish reference chart lost its predictive validity for ND. LGA, on the other hand, is associated with a tendency to decrease the risk of neonatal death.

No significant influence of the type of hypertensive disorder on stillbirth was found in the multivariate model. The strongest predictor of stillbirth was prematurity and SGA on the Intergrowth chart (OR 6.78). 

A larger number of factors affected the occurrence of CNO, stillbirth, and ND. The multivariate model for CNO showed a significant influence of maternal age, hypertension, parity, obesity, and growth abnormalities (LGA and SGA by Intergrowth). Prematurity was the strongest risk factor of CNO (OR 16.3), followed by placental abruption (OR 4.3) and HELLP syndrome (OR 2.54).

## 4. Discussion

This study aimed to establish an association between different hypertensive disorders of pregnancy, growth abnormalities, and neonatal outcome and confirm previously established risk factors as well as identify new risk factors of stillbirth, neonatal death, and composite neonatal outcome. Our data show that prematurity is the strongest risk factor of all adverse perinatal outcomes. That is a known fact for all complications of pregnancy [23,24]. For this reason, to assess the influence of other risk factors in the multivariate model, a separate logistic model was performed using a backward stepwise validation method to adjust for gestational age. In our study, superimposed preeclampsia has a strong association with adverse outcomes, especially neonatal mortality related to the presence of neonatal growth restriction [25]. Both SGA and NGR are higher with preeclampsia but depend on growth charts used to define abnormal growth [5,26].

The relationship between preeclampsia and SGA has been studied previously, but not in the aspect of growth charts used and defining newborn growth restriction among SGA neonates. The most studied pathology is preeclampsia which is primarily associated with placental lesions [4,12,25,26]. A significant inverse association was established between gestational age at delivery and small-for-gestational-age in a large multicenter study of singleton pregnancies [26]; our study confirms this association. The prevalence of SGA with PE was 82%, 47%, and 30% in those delivered at less than 34 weeks, between 34 and 37 weeks, and greater or equal to 37 weeks, respectively. SGA frequency in pregnancies without PE was significantly lower and was 44%, 21%, and 8%, respectively [13]. The Association of CH and CH with PE and SGA has also been studied [26]. In pregnancies with CH, compared to those without CH, there is a two-fold increase in the incidence of SGA neonates and a 10-fold increase in the incidence of PE. In pregnancies that did not develop PE, the incidence of SGA < 5th percentile was 10.3% in those with CH and 5.7% in those without CH, whereas in pregnancies that developed PE, the incidence of SGA was about 20% in both groups. Consequently, the increase in SGA in pregnancies with CH is partly due to the association of CH with a high incidence of superimposed PE, but it is primarily the consequence of the disease itself [14].

The average age of patients with CH and PE is higher than in other groups. Advanced maternal age is a known factor associated with higher maternal complications, including HPD [25,26]. Mean hospitalization time is longer for HPD than the control group and is the longest in the CH group with superimposed PE and correlates with the severity of the condition. The occurrence of placental abruption is a significant risk factor of all adverse outcomes [27,28]. Especially in pregnancies with preeclampsia, this complication doubles with the presence of PE and increases six times in comparison to the control group. Previous studies confirm that HDP, especially complicated with PE, correlated with increased placental abruption rate resulting in adverse perinatal outcomes [29,30,31].

Our study shows the importance of choosing a growth chart that best correlates with adverse outcomes. Both the Fenton growth chart and Polish reference chart are biased for not excluding birthweights of neonates from complicated pregnancies, especially by HDP [6,19]. This could be the strongest factor responsible for why standard charts, such as the Intergrowth chart, perform better in predicting poor outcomes. Interestingly, before adjusting for gestational age, the Polish reference growth chart was the only independent risk factor of stillbirth [6]. These comparisons perfectly illustrate how the methodology used to create growth charts can affect the obtained results [8,32,33]. Researchers should have extreme caution when interpreting the results generated on different types of growth charts. Only the standard charts allow comparing the effect of pathology on the outcome of pregnancy [34].

The consensus-based definition of newborn growth restriction includes prenatal diagnosis of abnormal growth and maternal placental disease [35]. The percentage of diagnosis of newborn growth restriction varies depending on the growth chart used. The standardized growth chart (Intergrowth) in comparison to the reference chart (Fenton) was a much stronger predictor of the risk of neonatal death [18,19]. Perhaps this is because standardized charts exclude maternal risk factors of abnormal growth [7]. This is a novel finding, which validates the importance of inclusion in the developed consensus definition of prenatal diagnosis of growth restriction and the presence of maternal hypertension [5].

Perinatal mortality is the most severe complication and was directly related to early preterm labor (<34 Hbd), confirmed by previous data [36,37,38]. Multiparity was associated with an increased neonatal death rate, but it reduced the risk of neonatal morbidity. Similar results were shown in studies that found nulliparous women compared to multiparous to have better neonatal outcomes even in the presence of a hypertensive disorder, SGA, and advanced maternal age [26]. The highest risk of stillbirth was in the GH group, and the mortality of newborns was strongly related to the presence of superimposed PE. The highest neonatal mortality was in CH with PE group (30.12 OR) rather than in PE (OR 12.92) and CH without PE (OR 8.08). The significant impact of preeclampsia as an independent risk factor of neonatal mortality was shown in the literature [39]. HELLP syndrome was found to be a severe risk factor for stillbirth [40].

The occurrence of neonatal complications was associated with advanced maternal age [26,41]. As in perinatal mortality, we observed an association between HPD and an increased incidence of CNO. The highest risk of neonatal complications was in groups with PE. HELLP syndrome was also associated with increased morbidity in the newborn. Pregnancy cholestasis, gestational diabetes, and obesity did not affect perinatal mortality [42]. However, they were associated with a slight increase in the incidence of CNO [43,44]. Nevertheless, a few studies were published showing that appropriate pregnancy management (glucose level in diabetes, low energy intake in obese women, and total bile acids monitoring in cholestasis) decreases the risk of perinatal outcomes, including neonatal death [45,46,47,48,49]. This could be a source of bias in our study because our hospital is a tertiary high-risk pregnancy ward with high-quality outpatient high-risk care. 

This study has several strengths and limitations. It is a retrospective study that always carries a specific risk of bias discussed in the methodology section of the study. The diagnosis of fetal growth restriction needed for defining newborn growth restriction was based on a diagnosis of FGR in the medical records and not an ultrasound database. In the initial analysis, we observed a huge impact of gestational age at delivery on neonatal outcomes, neonatal death, and stillbirth. Therefore, a second logistic model was required to show the influence of hypertension disorders and growth abnormalities on neonatal outcomes. 

This is the first study to analyze the relationship of outcome and growth restriction of the newborn based on the consensus definition published in 2018 by Beune et al. [4]. We have also shown the impact of applying a standardized growth chart in pregnancies complicated by hypertensive disorders. Standard growth charts perform better for the prediction of outcomes. The large size of this cohort and the application of a standardized international growth chart allow for the generalizability of these data.

## 5. Conclusions

Hypertensive disorders are significantly associated with growth abnormalities and are an independent risk factor of adverse outcomes. The presence of newborn growth restriction is strongly associated with the risk of neonatal death. This relationship is much stronger than that of SGA only. The choice of growth chart has a substantial effect on the percentage of diagnosis of SGA and NGR. Future studies of long-term outcomes of growth-restricted newborns diagnosed based on the consensus definition are essential for further validating the definition.

## Figures and Tables

**Table 1:** The characteristics of birth-weight charts used in the study.

Name of Chart	Citation	Methodology
Polish Reference chart	Kajdy, A.; Modzelewski, J.; Filipecka-Tyczka, D.; Pokropek, A.; Rabijewski, M. Development of Birth Weight for Gestational Age Charts and Comparison with Currently Used Charts: Defining Growth in the Polish Population. *The Journal of Maternal-Fetal & Neonatal Medicine* **2019,** 1–8 [6]	Based on a single-center birthweight analysis
Intergrowth	Garza, C. The INTERGROWTH-21st Project and the Multicenter Growth Reference Study: Enhanced Opportunities for Monitoring Growth from Early Pregnancy to 5 Years of Age. *Breastfeeding Medicine* **2014**, *9*, 341–344 [18]	International growth chart of birthweight
Fenton	Fenton, T.R.; Kim, J.H. A Systematic Review and Meta-Analysis to Revise the Fenton Growth Chart for Preterm Infants. *BMC Pediatr* **2013**, *13*, 59 [19]	Metaanalysis of birthweight charts from different countries; recommended chart by Polish Neonatology Society

**Table 2:** Characteristics of the population.

	GH Group 1 *n* = 2060 (%) A ± SD	CH Group 2 *n* = 542 (%) A ± SD	PE Group 3 *n* = 434 (%) A ± SD	CH with PE Group 4 *n* = 72 (%) A ± SD	Control Group *n* = 60,393 (%) A ± SD	ALL *n* = 63,501 (%) A ± SD	*p*-Value
Age [years]	31.4 ± 4.6	33.5 ± 4.9	31.2 ± 4.7	32.7 ± 5.4	31.2 ± 4.3	31.2 ± 4.3	<0.001
Education							<0.001
Primary	130 (6)	46 (9)	45 (10)	8 (11)	4044 (7)	4273 (7)	
Secondary	306 (15)	98 (18)	64 (15)	16 (23)	6638 (11)	7122 (11)	
Vocational	21 (1)	18 (3)	5 (1)	1 (1)	588 (1)	633 (1)	
Higher	1603 (78)	380 (70)	320 (74)	47 (65)	49,123 (81)	51,473 (81)	
Duration of hospitalization [days]	6.97 ± 5.2	7.1 ± 5.6	11.4 ± 8.0	13.4 ± 9.4	4.5 ± 5.2	4.7 ± 4.0	<0.001
Pregnancy outcomes:							
DM	170 (8)	61 (11)	36 (8)	6 (8)	3808 (6)	4081 (6)	<0.001
GDMG1	74 (4)	45 (8)	13 (3)	7 (10)	1227 (2)	1366 (2)	<0.001
GDMG2	16 (1)	5 (1)	2 (0.5)	0	168 (0.3)	191 (0.3)	<0.001
Undiagnosed and untreated DM							
HELLP syndrome	55 (3)	4 (1)	15 (4)	2 (3)	0	76 (0.1)	<0.001
Pregnancy cholestasis	23 (1)	8 (1.5)	4 (1)	0	746 (1)	781 (1)	0.62
Placental abruption	34 (2)	6 (1)	19 (4)	2 (3)	349 (0.6)	410 (0.6)	<0.001
Obesity	205 (10)	93 (17)	29 (7)	12 (17)	876 (1.5)	1215 (2)	<0.001
Maternal smoking	7 (0.3)	11 (2)	6 (1)	1 (1)	284 (0.5)	309 (0.5)	<0.001
Obstetric interview:							
Gestational week at delivery	38.4 ± 1.7	37.9 ± 2.4	35.9 ± 3.3	35.2 ± 3.4	39.0 ± 1.6	39.0 ± 1.6	<0.001
<34 hbd	45 (2)	29 (5)	86 (20)	18 (25)	609 (1)	787 (1)	
35–37 hbd	400 (19)	126 (23)	206 (47)	37 (51)	5752 (10)	6521 (10)	
>37 hbd	1615 (79)	387 (72)	142 (33)	17 (24)	54,032 (89)	56,193 (89)	<0.001
Parity							<0.001
1	1291 (63)	242 (45)	311 (72)	39 (54)	30,700 (51)	32,583 (51)	
2	579 (28)	202 (37)	93 (21)	21 (29)	22,416 (37)	23,311 (37)	
3	138 (7)	69 (13)	26 (6)	8 (11)	5478 (9)	5719 (9)	
>3	52 (2)	29 (5)	4 (1)	4 (6)	1799 (3)	1888 (3)	
Delivery:							
Labor induction	847 (41)	184 (34)	139 (32)	14 (19)	10,149 (17)	11,333 (18)	<0.001
CS	926 (45)	278 (51)	324 (75)	60 (83)	17,397 (29)	18,985 (30)	<0.001
CS for fetal distress	291 (14)	66 (12)	100 (23)	14 (19)	3432 (6)	3903 (6)	<0.001
Operational delivery for fetal distress	41 (2)	11 (2)	4 (1)	2 (3)	855 (1)	913 (1)	0.13
Placental abruption	34 (2)	6 (1)	19 (4)	2 (3)	349 (0.6)	410 (0.6)	<0.001
Infant outcomes:							
Male	1035 (50)	308 (57)	222 (51)	36 (50)	30,806 (51)	32,407 (51)	0.1
Female	1025 (50)	234 (43)	212 (49)	36 (50)	29,587 (49)	31,094 (49)	
Infant length [cm]	53.7 ± 3.6	53.3 ± 4.4	49.2 ± 6.2	48.7 ± 6.7	54.4 ± 3.7	54.3 ± 3.1	<0.001
Infant weight [g]	3285 ± 607	3220 ± 669	2530 ± 880	2464 ± 923	3432 ± 490	3417 ± 508	<0.001
Polish scale							<0.001
AGA	1510 (73)	425 (78)	268 (62)	44 (61)	48,451 (80)	50,698 (80)	
LGA	232 (11)	54 (10)	19 (4)	5 (7)	6080 (10)	6390 (10)	
SGA	318 (16)	63 (12)	147 (34)	23 (32)	5862 (10)	6413 (10)	
Fenton scale							<0.001
AGA	1683 (82)	467 (86)	314 (72)	54 (75)	53,224 (88)	55,752 (88)	
LGA	179 (8)	44 (8)	17 (4)	4 (6)	3948 (7)	4192 (7)	
SGA	198 (10)	31 (6)	103 (24)	14 (19)	3221 (5)	3567 (5)	
Intergrowth scale							<0.001
AGA	1455 (71)	393 (72)	307 (71)	47 (65)	45,358 (75)	47,560 (75)	
LGA	485 (24)	129 (24)	41 (9)	12 (17)	13,628 (23)	14,295 (22)	
SGA	120 (6)	20 (4)	86 (20)	13 (18)	1407 (2)	1646 (3)	
CND	8 (0.4)	6 (1)	7 (2)	2 (3)	148 (0.3)	171 (0.3)	<0.001
Stillbirth	8 (0.4)	2 (0.4)	2 (0.5)	0	97 (0.2)	109 (0.2)	0.048
ND	0	4 (1)	5 (1)	2 (3)	51 (0.1)	62 (0.1)	<0.001
NGR Fenton	156 (8)	29 (5)	97 (22)	14 (19)	727 (1)	1023 (2)	<0.001
NGR Intergrowth	120 (6)	23 (4)	91 (21)	13 (18)	408 (1)	655 (1)	<0.001
CNO	651 (32)	174 (32)	240 (55)	41 (57)	11,354 (19)	12,460 (20)	<0.001
Apgar in 5th minute ≤ 7	16 (1)	9 (2)	17 (4)	4 (6)	260 (0.4)	306 (0.5)	<0.001
RDS	61 (3)	36 (7)	93 (21)	24 (33)	1161 (2)	1375 (2)	<0.001
IVH grade III or IV	1 (0.1)	1 (0.2)	1 (0.2)	0	12 (0.02)	15 (0.02)	0.005
Neonatal sepsis	7 (0.3)	7 (1)	8 (2)	2 (3)	150 (0.3)	174 (0.3)	<0.001
Leukomalacia	1 (0.1)	0	1 (0.2)	0	7 (0.01)	9 (0.01)	0.03
Hypothermia	1 (0.1)	1 (0.2)	1 (0.2)	0	35 (0.1)	38 (0.06)	0.45
Neonatal seizures	0	0	0	0	24 (0.04)	24 (0.04)	0.66
NICU admission	428 (21)	132 (24)	204 (47)	39 (54)	8024 (13)	8827 (14)	<0.001
NICU stay [days]	1.5 ± 5.7	9.5 ± 18.3	2.4 ± 9.4	11.7 ± 22.6	0.7 ± 4.0	0.8 ± 4.5	<0.001

**Table 3:** Percentage of SGA defined as NGR by consensus definition [5] depending on the chart used (Fenton vs. Intergrowth).

		Definitions of Growth Restriction in the Newborn			Difference in % of Defined Neonates
	SGA, Birthweight < 10 Centile (*n*)	NGR, Birthweight < 3 Centile (*n*)	NGR, Consensus 3/5 (Beune)	NGR Consensus [Weight < 3 + 3/5 Conditions *	SGA vs. NGR
Fenton	3567	787	236	1023	29%
Intergrowth	1646	376	279	655	40%

* NGR definition: birth weight less than the third percentile on population-based charts or at least 3 out of 5 of the following: birth weight < 10th percentile on population-based charts, head circumference < 10th percentile, length < 10th percentile, prenatal diagnosis of fetal growth restriction, maternal pregnancy information regarding hypertension and/or preeclampsia.

**Table 4:** Logistic regression for neonatal outcomes.

	Stillbirth OR (95% CI)	ND OR (95% CI)	CNO OR (95% CI)
Maternal age	0.7 (0.29–1.72)	0.99 (0.94–1.05)	**1.015 (1.01–1.02)**
Parity	1.18 (0.97–1.43)	**1.33 (1.07–1.65)**	**0.74 (0.72–0.76)**
Gestational week at delivery			
<34 hbd	**231.2 (144–371.2)**	**742.7 (318.2–1734)**	**19.0 (15.9–22.7)**
34–37 hbd	**6.84 (3.75–12.5)**	**4.32 (1.08–17.28)**	**0.215 (0.21–0.22)**
>37 hbd	1.0	1.0	1.0
GH	**2.37 (1.15–4.87)**	-	**1.94 (1.77–2.14)**
CH	2.19 (0.54–8.89)	**8.08 (2.92–22.3)**	**1.95 (1.63–2.34)**
PE	2.75 (0.68–11.19)	**12.92 (5.15–32.4)**	**5.15 (4.26–6.23)**
CH with PE	**-**	**30.12 (7.22–125.7)**	**5.43 (3.4–8.67)**
HELLP Syndrome	**7.81 (1.08–56.68)**	-	**8.91 (5.49–14.46)**
Pregnancy cholestasis	-	-	**1.41 (1.2–1.66)**
DM			
GDMG1	0.7 (0.29–1.72)	1.01 (0.37–2.77)	**1.13 (1.04–1.22)**
GDMG2	0.85 (0.21–3.44)	-	**1.16 (1.02–1.32)**
Undiagnosed DM	3.07 (0.43–22.2)	-	1.16 (0.82–1.63)
Obesity	1.96 (0.72–5.32)	1.71 (0.42–7.0)	**1.54 (1.36–1.75)**
Fenton scale			
AGA	1.0	1.0	1.0
LGA	1.23 (0.53–2.83)	0.29 (0.04–2.1)	**1.19 (1.1–1.28)**
SGA	**9.26 (6.19–13.83)**	**5.16 (2.88–9.25)**	**1.76 (1.63–1.9)**
Intergrowth scale			
AGA	1.0	1.0	1.0
LGA	0.59 (0.31–1.12)	**0.2 (0.06–0.64)**	0.97 (0.93–1.02)
SGA	**17.2 (11.38–26)**	**5.34 (2.62–10.87)**	**2.29 (2.07–2.54)**
Polish scale			
AGA	1.0	1.0	1.0
LGA	0.94 (0.43–2.06)	0.37 (0.09–1.52)	**1.09 (1.02–1.16)**
SGA	**5.8 (3.92–8.61)**	**3.15 (1.8–5.52)**	**1.47 (1.39–1.56)**
NGR Fenton	**7.7 (4.21–14.06)**	**10.6 (5.2–21.5)**	**2.5 (2.2–2.84)**
NGR Intergrowth	**13.43 (7.49–24.1)**	**19.1 (9.65–37.7)**	**3.23 (2.76–3.77)**
Placental abruption	**16.3 (8.44–31.48)**	**31 (15.66–61.5)**	**6.72 (5.5–8.2)**

* statistical significant data were bolded.

**Table 5:** Multivariate logistic regression for neonatal outcomes.

	aOR (95% CI)	*p*-Value
Stillbirth (without NGR)		
Gestational week at delivery		
<34 hbd	166 (101.8–270.6)	<0.001
34–37 hbd	5.72 (3.12–10.5)	<0.001
Intergrowth SGA	6.78 (4.29–10.72)	<0.001
ND (with gestational age)		
Gestational week at delivery		
<34 hbd	699 (294–1606)	<0.001
34–37 hbd	4.21 (1.05–16.9)	0.04
Polish SGA	2.37 (1.3–4.31)	0.005
ND (without gestational age)		
Parity	1.37 (1.11–1.68)	0.003
Intergrowth LGA	0.19 (0.06–0.6)	0.006
Intergrowth SGA	0.36 (0.07–1.99)	0.24
Intergrowth NGR	22.1 (3.97–123)	<0.001
PE	5.32 (1.82–15.6)	0.002
CH	6.2 (2.14–17.91)	0.001
CH with PE	12.3 (2.55–59.1)	0.002
CNO		
Age	1.04 (1.03–1.05)	<0.001
Parity	0.67 (0.65–0.69)	<0.001
Obesity	1.29 (1.13–1.48)	<0.001
Gestational week at delivery		
<34 hbd	16.3 (13.6–19.6)	<0.001
34–37 hbd	1.7 (1.6–1.8)	<0.001
Placental abruption	4.3 (3.45–5.36)	<0.001
GH	1.62 (1.47–1.8)	<0.001
PE	2.34 (1.9–2.89)	<0.001
CH	1.58 (1.3–1.93)	<0.001
CH with PE	2.32 (1.36–3.95)	0.002
HELLP syndrome	2.54 (1.5–4.33)	0.001
Intergrowth LGA	1.07 (1.01–1.1)	0.01
Intergrowth SGA	1.71 (1.53–1.91)	<0.001

## Data Availability

The datasets analyzed during the current study are available from the corresponding author on reasonable request.
